# MMP11 is associated with the immune response and immune microenvironment in EGFR-mutant lung adenocarcinoma

**DOI:** 10.3389/fonc.2023.1055122

**Published:** 2023-01-23

**Authors:** Lu Bai, Ran Huo, Guotao Fang, Tiantian Ma, Yanhong Shang

**Affiliations:** Hebei Key Laboratory of Cancer Radiotherapy and Chemotherapy, Department of Medical Oncology, Affiliated Hospital of Hebei University, Baoding, China

**Keywords:** matrix metalloproteinase 11 (MMP11), lung adenocarcinoma, epidermal growth factor receptor (EGFR), immune microenvironment, immunotherapy

## Abstract

**Background:**

High expression of matrix metalloproteinase-11 (MMP11) is associated with various tumors and immune microenvironments. Conversely, poor response to immunotherapy in epidermal growth factor receptor (EGFR)-mutant lung adenocarcinoma (LUAD) patients is closely related to the characteristics of immune microenvironment.

**Methods:**

The Cancer Genome Atlas (TCGA)-LUAD database and our gathered clinical LUAD samples were used to examine the relationship between MMP11 expression and EGFR mutation. Then the correlation between MMP11 and immune response and the difference of immune cell infiltration in different groups were analyzed. Compared the differences in the immune microenvironment between the MMP11-positive and MMP11-negative expression groups using immunohistochemistry (IHC) and multiplex immunohistochemistry.

**Results:**

The expression of MMP11 in samples with exon 19 deletions, exon 21 L858R or *de novo* exon 20 T790M mutations was higher than wild type, but there was no difference between the samples with uncommon mutation and the wild-type. The high MMP11 expression group had a higher Tumor Immune Dysfunction and Exclusion (TIDE) score. Pathways associated with enrichment in the extracellular matrix (ECM) were the main biological functions of differential genes between the high and low MMP11 groups. The IHC score of MMP11 in the EGFR-mutant group was higher than in the EGFR-wild group. In TCGA-LUAD, the high MMP11 group had a lower proportion of T cell CD8+ and NK cells activated. In the clinical samples, the infiltration levels of T cell CD8+ and NK cells in the tumor parenchyma of EGFR-mutant LUAD was lower in the MMP11-positive than in the MMP11-negative group. The expression levels of tumor cell PD-L1 were higher in the MMP11-positive expression group than in the MMP11-negative expression group, and the proportion of PD1+CD8+ T cells infiltrated was reduced in the MMP11-positive group compared to the MMP11-negative group.

**Conclusions:**

High expression of MMP11 was associated with EGFR mutations. Patients with EGFR-mutant LUAD with high expression of MMP11 responded poorly to immunotherapy, and the percentage of T cell CD8+ and NK cells in immune cell infiltration was lower in MMP11. Consequently, MMP11 is related to the immunological microenvironment of EGFR-mutant lung adenocarcinoma, which may be a predictor of possible immunotherapeutic response.

## 1 Introduction

Lung cancer is one of the major killers of human cancers, with a high mortality rate, and lung adenocarcinoma (LUAD) accounts for most cases (1). Tumor initiation and development are closely correlated with the epidermal growth factor receptor (EGFR), the most prevalent mutation driver gene in LUAD (2). The primary therapy for LUAD with EGFR gene mutations is epidermal growth factor receptor tyrosine kinase inhibitors (EGFR-TKIs) (3). However, a new obstacle has emerged in treating these patients: EGFR-TKI resistance can occur either primary or secondary, regardless of the generations (4, 5). Currently, immune checkpoint inhibitors (ICIs) have significantly improved the treatment of LUAD (6). According to multiple clinical studies using ICIs to treat advanced lung cancer, immunosuppressive agents combination chemotherapy or single-agent immunotherapy treatments can increase the survival chances of patients for five years by roughly 30% compared to chemotherapy alone. However, EGFR-mutant LUAD responds to ICIs quite differently: EGFR-mutant patients in both a pooled second-line research of immune monotherapy and a first-line study of immune monotherapy did not benefit while suffering substantial toxicities. EGFR mutations predicted primary resistance to ICIs and hyperprogression to immune monotherapy (7, 8). However, patients with EGFR mutations are not without immunotherapy opportunities. In the IMpower150 study, patients after resistance to EGFR-TKIs therapy can already benefit from immune combination therapy (9). The clinical trial result suggested that “primary resistance” to ICIs in EGFR-mutated NSCLC is not absolute and possibly reversible. *In vitro* trials have shown that the immunosuppressive response in EGFR-mutant NSCLC can be improved by certain interventions (10, 11). Consequently, it is crucial to thoroughly research the characteristics of the immune microenvironment of EGFR-mutant LUAD, identify the critical elements causing immune escape in this population of patients, and consider new treatment modalities and strategies to overcome drug resistance.

Catabolism of cell adhesion factors and modification of cell-cell contacts are the two main regulatory activities of matrix metalloproteinases (MMPs), zinc-related peptide endonucleases (12–14). MMPs have an immunosuppressive regulatory function, are intimately associated with the immunological microenvironment of tumors, and may play a role in the immune escape of malignancies (15). Matrix metalloproteinase-11 (MMP11), a member of MMPs, also known as stromelysin-3, was first identified in breast cancer (16). MMP11 is expressed in many tumors, such as oral cancer, lung cancer, esophageal cancer, pancreatic cancer, invasive meningioma, ovarian cancer, and colon cancer, while expression in normal tissues is rare (17). Through bioinformatics analysis, *in vitro* cellular assays, and tumorigenic assays in mice, Haoran Yang et al. (18) discovered that MMP11 is highly expressed in LUAD tissues compared to normal tissues. They also discovered that the absence of MMP11 severely inhibited the proliferation, migration, and invasion of LUAD cells in contrast to xenograft MMP11 antibodies that impede the expansion and migration of various human-derived lung cancer cell lines *in vitro* cell experiments. Using the MMP11 antibody led to a considerable reduction of tumor growth in a xenograft model. Strong positive expression of MMP11 was significantly and negatively related to overall survival in a proteogenomic landscape investigation of lung cancer patients in East Asia (19). Furthermore, it is yet unknown whether MMP11 is related to immune escape in lung cancer, particularly in individuals with EGFR mutation-positive LUAD.

To generate new concepts for immunotherapy in this population of patients, we investigated the relationship between MMP11 and EGFR mutation in LUAD patients herein, followed by the relationship between MMP11 and the immune microenvironment in EGFR-mutant LUAD patients.

## 2 Materials and methods

### 2.1 Data source

The Oncomine database (http://www.oncomine.org/) was searched for MMP11 gene analysis type: cancer vs. normal analysis was used to examine MMP11 expression in various cancers (20). The TIMER database (https://cistrome.shinyapps.io/timer/) (21) was used to assess further the expression of MMP11 in various malignancies (21). MMP11 expression in various tumors and subtypes was examined. For LUAD patients, gene expression data (HTSeq-FPKM) were acquired from The Cancer Genome Atlas database (TCGA, https://gdc.cancer.gov/) (22). Data on gene variations and clinical profiles related to TCGA lung cancer patients were provided from the UCSC Xena database (https://xenabrowser.net/datapages/) (23). EGFR mutation subtypes information for the samples was obtained from cBioPortal (https://www.cbioportal.org/) (24).

### 2.2 Data processing

The RNA sequencing profile downloaded in fragments per kilobase million (FPKM) format from the TCGA-LUAD database was converted into transcripts per million (TPM) format, and log2 was transformed. All samples were screened to keep samples with clinical information and eliminate duplicates. Based on EGFR mutation status, lung cancer samples were separated into mutated and wild groups, and the variation in MMP11 expression at the mRNA level was examined. To distinguish between MMP11 expression levels that are high and low, the median of MMP11 mRNA expression in LUAD was employed as a cut-off. Differential mRNA expression was studied using the Limma package (version 3.40.2) of the R program. Adjusted P < 0.05 and log2 (fold change) > 1 or log2 (fold change) < -1” was defined as the threshold mRNA differential expression screen. Potential mRNAs were examined using the ClusterProfiler tool for the Gene Ontology/Kyoto Encyclopedia of Genes and Genomes (GO/KEGG) analysis to understand the function of target genes in the immunological microenvironment. The STRING (http://string-db.org) database was used to identify Protein-Protein Interaction Networks (PPIs) between the 50 genes with the most significant variability and EGFR (25). PPIs with confidence values of ≥0.4 were retained and visualized for analysis using the igraph package (version 1.2.6), ggraph package (version 2.0.5). The correlation of MMP11 in the TCGA-LUAD dataset with the dysfunction of tumor-infiltrating cytotoxic T lymphocytes (CTL) and the rejection of CTL by immunosuppressive factors was explored using Tumor Immune Dysfunction and Exclusion (TIDE, http://tide.dfci.harvard.edu/) (26). Then, the prospective immunotherapeutic response of patients in the high or low MMP11 expression groups in EGFR-mutant LUAD was predicted using TIDE scoring. Using the immunedeconv R package and the CIBERSORT algorithm (https://cibersort.stanford.edu/), RNA-seq data from 511 tumors in the TCGA-LUAD database were converted into 22 immune cells.

### 2.3 Sample collection and subgroups

Patients (regardless of gender and age) with the first diagnosis of wild-type and mutated EGFR from July 2019 to July 2021 were collected from the Department of Medical Oncology, Affiliated Hospital of Hebei University. **Supplementary Table 1** shows the inclusion and exclusion criteria for sample collection. This study was approved by the ethics committee of the Affiliated Hospital of Hebei University, and patients signed an informed consent form prior to enrollment.

### 2.4 Immunohistochemistry

The protein expression of MMP11 in LUAD tissue was measured according to standard immunoperoxidase staining procedures. The immunohistochemistry (IHC) score for MMP11 was assessed by two independent pathologists. The percentage of immunohistochemically stained cells was scored as follows: percentage of stained cells 0%–25% was scored as 1, 26%–50% was scored as 2, 51%–75% was scored as 3, and 75%–100% was scored as 4. The intensity of immunohistochemical staining was scored as 0 for no staining, 1 for weak staining, 2 for moderate staining, and 3 for strong staining. The percentage of positive tumor cells and stain intensity were multiplied to generate a weighted score for each case. All cases were divided into two groups according to the immunohistochemical score of MMP11. MMP11-positive was defined as an immunohistochemical score ≥ 6; MMP11-negative was defined as an immunohistochemical score < 6.

### 2.5 Multiplex immunohistochemistry methods

Multiplex immunohistochemistry techniques were used to determine the degree of immune cell infiltration in the tumor parenchyma of a single EGFR mutant sample. This covers the expression levels of CD8+ T cells, CD3+ T cells, M1 macrophages, macrophage M2, PD-L1 on tumor cells, PD-1 on immune cells, and NK cells.

### 2.6 Statistical methods

The data processing described above was analyzed using R (version 3.6.3) and SPSS (version 26.0), and the significance of two groups of samples was tested using the Wilcoxon test. The significance of three groups of samples was tested using the Kruskal-Wallis test. Fisher’s exact probability method was used to analyze MMP11 expression, and clinical characteristics in clinical samples were collected using multiplex immunohistochemistry for EGFR mutations. The correlation between all was considered statistically significant at P < 0.05. Statistical identifiers: ns, P ≥ 0.05; *, P < 0.05; **, P < 0.01; ***, P < 0.001.

## 3 Results

### 3.1 Differences in MMP11 expression between the EGFR mutation group and wild group in LUAD patients

**Figure 1:**
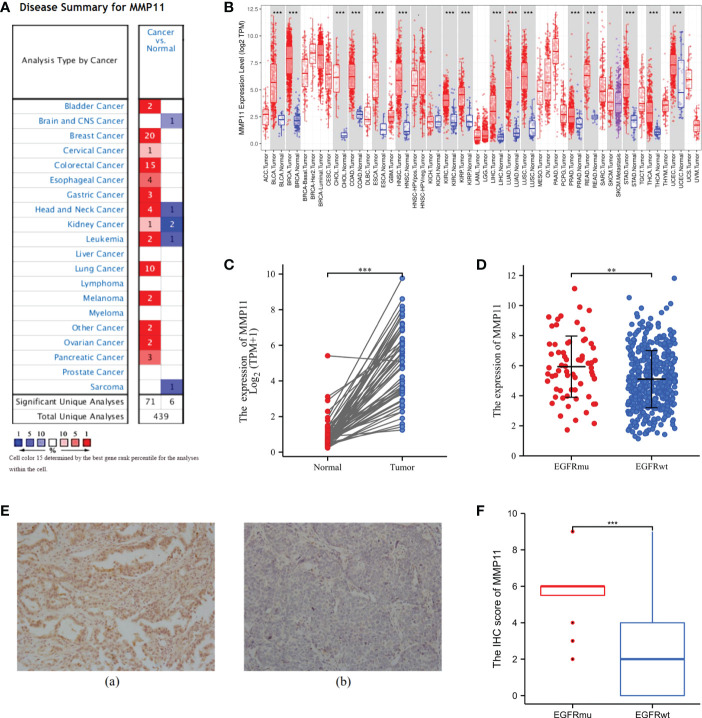
Differential expression of MMP11. **(A)** Differential expression of MMP11 in different tumor tissues and normal tissues in the Oncomine database; **(B)** Differential expression of MMP11 in different tumor tissues and normal tissues in the TIMER database; **(C)** Differential expression of MMP11 in LUAD tissues and paraneoplastic tissues in TCGA database; **(D)** Differences in MMP11 expression between EGFR-mutant group and EGFR-wild group in TCGA-LUAD database; **(E)** MMP11 immunohistochemical results of EGFR-mutant LUAD and EGFR wild-type LUAD tumor tissues: Positive MMP11 immunohistochemical staining (a) vs. negative staining (b) (400X); **(F)** Differences in IHC scores of MMP11 in LUAD tissues from EGFR-mutant group and EGFR-wild group; **P < 0.01; ***P < 0.001.

According to an analysis of MMP11 gene expression in various cancer types using the Oncomine database, MMP11 was highly expressed in bladder, breast, cervical, colorectal, esophageal, gastric, head, and neck tumors, kidney, leukemia, lung, lymphoma, melanoma, ovarian, and pancreatic cancers when compared to normal tissues (**Figure 1A**) (high expression in red, low expression in blue). Using the Oncomine database, we analyzed MMP11 expression in malignant tumors and distinct subtypes using the TIMER online platform (**Figure 1B**). We discovered that in LUAD, MMP11 expression was higher in tumor tissues than in normal tissues. MMP11 expression levels in lung cancer patients were non-significantly related to TNM stage, gender, race, age, or smoking (P > 0.05) but rather to their primary therapy outcome (**Supplementary Table 2**). Consequently, by downloading the RNAseq expression profiles of 57 pairs of matched lung cancer tissues from the TGCA database (**Figure 1C**), we evaluated the variations in MMP11 expression between LUAD tissues and paraneoplastic tissues and conducted statistical descriptions. The mean level of MMP11 (blue) in the paracancerous tissue group was 0.417 ± 0.54 with a median of 0.256, while the mean level of MMP11 (red) in the LUAD group was 3.57 ± 1.868 with a median of 3.712. MMP11 mRNA expression was higher in LUAD than in paracancerous tissue (P < 0.001).

In the following, to further analyze the differences in MMP11 expression at the mRNA level in lung adenocarcinoma tissues under different EGFR mutation conditions, we obtained 512 samples with known EGFR mutation conditions from the TCGA-LUAD dataset. We divided them into EGFR mutant-group and EGFR-wild group to compare the difference of MMP11 expression level. MMP11 expression was higher in lung cancer tissue than in normal tissue (normal, n = 59, yellow), regardless of EGFR mutation status, according to a comparison of 512 LUAD samples with identifiable EGFR mutations information in the TCGA database and 59 normal lung tissue samples. In LUAD, the median MMP11 expression was 5.860 in the EGFR-mutant group (EGFRmu, n = 66, red) and 4.913 in the EGFR-wild group (EGFRwt, n = 446, blue), and MMP11 expression at the mRNA level was significantly increased in the EGFR-mutant group (P < 0.05) (**Figure 1D**). Consequently, MMP11 expression may be related to the mutational status of EGFR. Later, we collected clinical samples and evaluated the differences in protein levels of MMP11 between the EGFR-mutant group and the wild group by means of IHC. Finally, 20 mutant-type EGFR samples and 17 wild-type EGFR samples were obtained. MMP11 was expressed in the cytoplasm, and positive staining was found in the cytoplasm (**Figure 1E**). Immunohistochemical results showed that the IHC score of MMP11 in the EGFR-mutant group was 5.45 ± 1.538, in the EGFR-wild group was 2.353 ± 2.523, and in the EGFR-mutant group was higher than that in the EGFR-wild group (P < 0.001) (**Figure 1F**).

### 3.2 Differential expression of MMP11 in LUAD samples with different EGFR mutation subtypes

**Figure 2:**
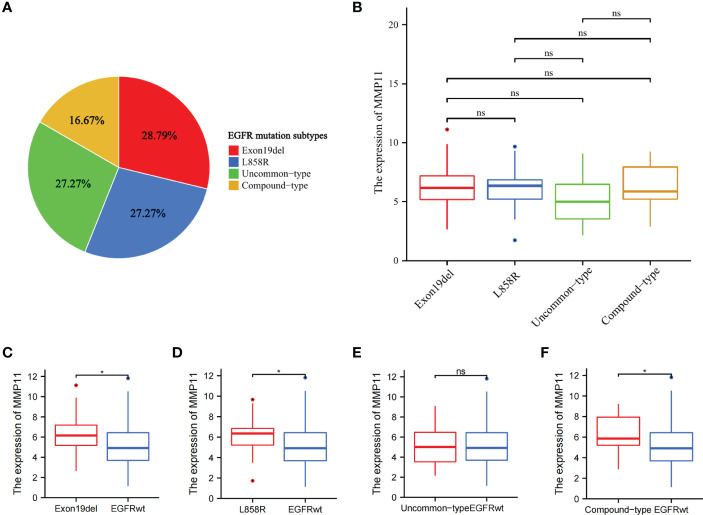
Differential expression of MMP11 between different EGFR mutation subtypes at the mRNA levels. **(A)** Proportion of EGFR-mutant samples by mutation subtypes among 66 EGFR-mutant LUAD, the Exon19del group is red, the L858R group is blue, the uncommon-type group is green and the compound-type group is yellow (pie chart; TCGA); **(B)** Differential expression of MMP11 in samples with different EGFR mutation subtypes at the mRNA level (TCGA); **(C)** Differences in MMP11 mRNA expression between the Exon19del and EGFR-wild groups (TCGA-LUAD); **(D)** Differences in MMP11 mRNA expression between the L858R and EGFR-wild groups (TCGA-LUAD); **(E)** Differences in MMP11 mRNA expression between the uncommon-type group and EGFR-wild groups (TCGA-LUAD); **(F)** Differences in MMP11 mRNA expression between the compound-type group and EGFR-wild groups (TCGA-LUAD); *P < 0.05; ns, no statistical significance.

**Figure 3:**
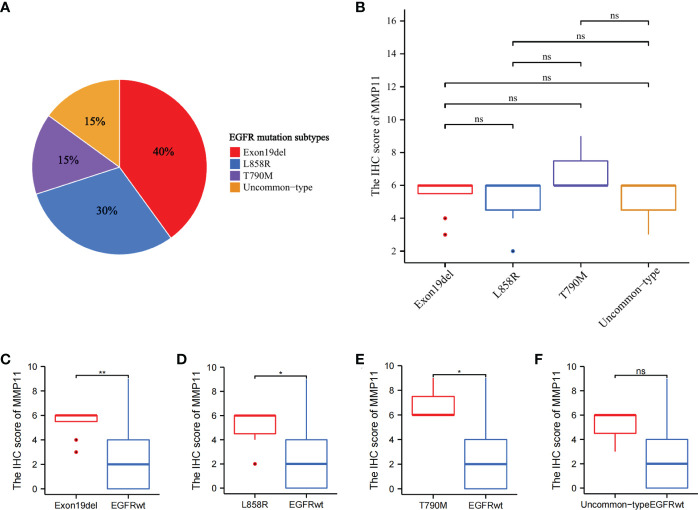
Differential expression of MMP11 between different EGFR mutation subtypes at the level of IHC scores. **(A)** Proportion of EGFR-mutant samples by mutation subtypes among 20 EGFR-mutant LUAD clinical samples, the Exon19del group is red, the L858R group is blue, the T790M group is purple, the uncommon-type group is yellow (pie chart); **(B)** The differences in MMP11 ICH scores between the Exon19del group (red), the L858R group (blue), the T790M group (purple), the uncommon-type group (yellow); **(C)** Differences in MMP11 IHC scores between the Exon19del and EGFR-wild groups; **(D)** Differences in MMP11 IHC scores between the L858R and EGFR-wild groups; **(E)** Differences in MMP11 IHC scores between the T790M and EGFR-wild groups; **(F)** Differences in MMP11 IHC scores between the uncommon-type group and EGFR-wild groups; *P < 0.05; **P < 0.01; ns, no statistical significance.

To further analyze the effect of different EGFR mutant isoforms on MMP11 expression, EGFR mutant samples in the TCGA-LUAD dataset were grouped according to EGFR mutant isoforms and the differences in the mRNA expression levels of MMP11 between the groups were compared. Information on EGFR mutation subtypes by sample can be found in the **Supplementary Data Sheet 1**. The 66 samples were divided into exon 19 deletions group (Exon19del), exon 21 L858R group (L858R), uncommon mutations group (uncommon-type) and compound mutations group (compound-type). Among the 66 EGFR mutant samples, there were 19 cases of Exon19del (red, 28.79%), 18 cases of L858R (blue, 27.27%), 18 cases of uncommon-type (green, 27.27%), and 11 cases of compound-type (yellow, 16.67%) (**Figure 2A**). We analyzed the differences in the expression of MMP11 in different EGFR mutation subtypes in TCGA-LUAD (**Figure 2B**), and the results showed that, in the expression level of MMP11, the mean level in the Exon19del group was 6.284 ± 2.076, 6.041 ± 1.929 in the L858R group, 5.194 ± 2.053 in the uncommon-type group, and 6.359 ± 2.076 in the compound-type group, with no significant differences between different EGFR mutation subtypes (P > 0.05). Considering the incomplete EGFR mutation subtypes in the TCGA-LUAD dataset and the small number of samples with the common drug-resistant mutation T790M mutation, we selected the collected clinical samples to further validate the relationship between MMP11 at the protein level and EGFR mutations. The clinical samples were divided into exon 19 deletions group (Exon19del), exon 21 L858R group (L858R), *de novo* exon 20 T790M group (T790M) and uncommon mutations group (uncommon-type). We then analyzed the difference in MMP11 expression between samples grouped by each EGFR mutation subtype and samples in the EGFR-wild group (**Figures 2C–F**). The mean level of MMP11 mRNA expression in the EGFR-wild group was 5.106 ± 1.901. We found that the expression levels of MMP11 were higher in the Exon19del, L858R, and compound-type group samples than in the EGFR-wild group samples, respectively (P < 0.05) (**Figures 2C, D, F**). There was no significant difference in MMP11 expression levels between the uncommon-type group and the EGFR-wild group (**Figure 2E**). Among the EGFR mutated clinical samples we collected, there were 8 cases in the Exon19del group (red, 40%), 6 cases in the L858R group (blue, 30%), 3 cases in the T790M group (green, 15%), and 3 cases in the uncommon-type group (yellow, 15%) (**Figure 3A**). We compared the differences in MMP11 ICH scores between the Exon19del group (red), the L858R group (blue), the T790M group (purple) and the uncommon-type group (yellow) (**Figure 3B**). The results showed that at the levels of MMP11 IHC scores, the mean value was 5.375 ± 1.188 for the Exon19del group, 5.375 ± 1.188 for the L858R group, 7.000 ± 1.732 for the T790M group and 5.000 ± 1.732 for the uncommon-type group, and between the groups There was no significant difference between the groups (P > 0.05). We also analyzed the differences in the IHC scores of MMP11 between samples from different EGFR mutation subtype groups and EGFR-wild group samples (**Figures 3C–F**). The mean value of MMP11 IHC scores in the EGFR-wild group was 2.353 ± 2.523. The results showed that samples in the Exon19del (P < 0.01), L858R (P < 0.05) and T790M (P < 0.05) groups had higher MMP11 IHC scores than samples in the EGFR-wild group, respectively (**Figures 3C–E**). However, samples in the uncommon-type group did not differ significantly from those in the EGFR-wild group at the MMP11 IHC score levels (**Figure 3F**).

### 3.3 Relationship between MMP11 and immune response

**Figure 4:**
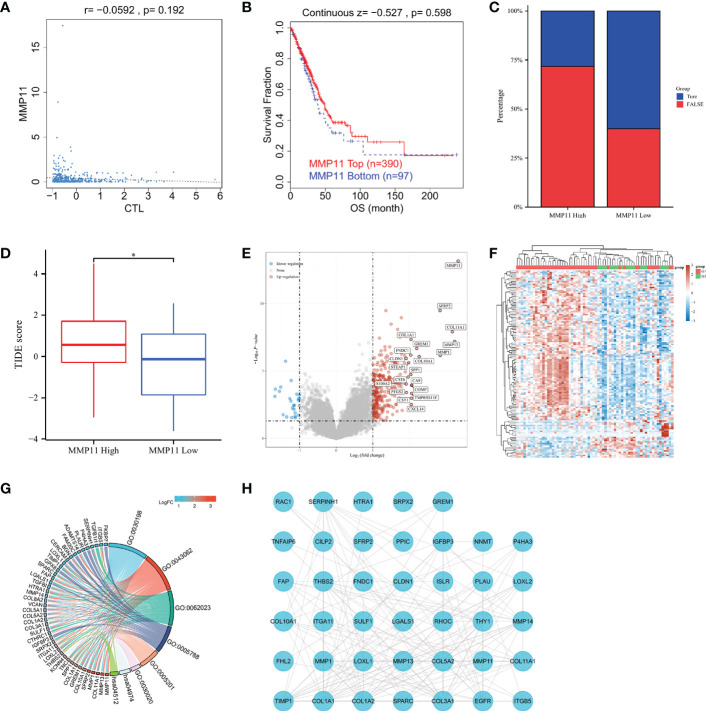
Relationship between MMP11 and immune response. **(A)** Correlation of MMP11 with cytotoxic T-cell level in TCGA-LUAD (TIDE); **(B)** Kaplan-Meier curves of survival ratios as a measure of the immunotherapeutic response (immune checkpoint blockade) between groups with high and those with low expression levels of MMP11 in TCGA-LUAD (TIDE); **(C)** Proportions of samples in EGFR mutant lung adenocarcinoma MMP11 high and low expression groups (histogram, TCGA); **(D)** Difference in TIDE scores between the high MMP11 expression group (red) and low expression group (blue) in EGFR-mutant LUAD (TCGA), *P < 0.05; **(E)** Differential gene analysis of EGFR-mutant LUAD MMP11 high and low expression groups; blue indicates downregulation; and red indicates upregulation; **(F)** Correlation heatamap of 50 upregulated genes and 50 downregulated genes; red indicates upregulation; blue indicates downregulation; the darker the color, the higher the correlation; **(G)** Functional enrichment analysis of the top 100 genes with the most significantly different trends of alteration; KEGG and GO; **(H)** PPIs of the 50 genes with the most significant differences in the EGFR mutant lung adenocarcinoma MMP11 high and low expression groups and EGFR.

We searched the TIDE website for MMP11 in the TCGA-LUAD dataset to explore the relationship between MMP11 and immune response. We found that MMP11 expression in lung adenocarcinoma did not correlate with cytotoxic T-cell level (P = 0.192) (**Figure 4A**). The Kaplan-Meier curve shows that MMP11 expression levels are not associated with clinical benefit of ICB treatment in lung adenocarcinoma (P = 0.598) (**Figure 4B**). We tried to explore the relationship between MMP11 and the immune response. Using a set of gene expression indicators, the TIDE score evaluates two pathways of tumor immune escape: malfunction of tumor-infiltrating cytotoxic T lymphocytes (CTL) and rejection of CTL by immunosuppressive agents (27). The higher the TIDE score, the worse the immunosuppressive efficacy and the shorter the survival after receiving immunosuppressive therapy. We next calculated the TIDE score of MMP11 in EGFR-mutant LUAD. In patients with EGFR-mutant LUAD (**Supplementary Data Sheet 2**). The results showed that only 13 (28.3%) of high-expression group responded to immunotherapy (True), and 33 (71.7%) did not respond (False). In contrast, 12 (60%) of the low expression group responded to immunotherapy (True), and 8 (40%) did not respond (False) (**Figure 4C**). The TIDE score was also significantly higher in the high MMP11 expression group (n = 46, red) than in the low MMP11 expression group (n = 20, blue), the differences were statistically significant (P < 0.05) (**Figure 4D**). We then analyzed the differential genes in the high- and low-expression groups of MMP11 in EGFR-mutant LUAD to explore the possible ways of their involvement in the immune response. The high MMP11 expression group (red, n=46) and the low MMP11 expression group (blue, n=20) were created from EGFR-mutant LUAD using the median expression of MMP11 mRNA in the TCGA-LUAD database as the standard cut-off point. In EGFR-mutant LUAD samples, the number of upregulated genes was 278, and the number of downregulated genes was 25 in the high MMP11 expression group compared to the low MMP11 expression group. SFRP2, COLF11A1, MMP13, MMP1, GREM1, FNDC1, COL10A1, SPP1, CLDNA, STEAP1, CST6, CA9, S100A2, COMP, PTGS2, TMPRSS11E, CST1, and CXCL14 were among the genes that were highly elevated in addition to MMP11 (**Figure 4E**). The heatmap of gene expression was plotted according to the 50 genes with the greatest differential change in expression 50 upregulated genes and 50 downregulated genes), with red denoting the upregulation trend and blue denoting the downregulation trend. This revealed that the G1 group had a higher concentration of upregulated genes, while the G2 group had a higher concentration of downregulated genes (**Figure 4F**). The top 100 differentially expressed genes with the greatest change were chosen for the GO/KEGG analysis. Different colors reflect the outcomes of differential enrichment logFC, which was conducted using the R packages “ClusterProfiler” (version:3.18.0), “GOplot” (version:1.0.2), and “ggplot2” (version:3.3.3). The findings demonstrated that in EGFR-mutant LUAD, the differentially expressed genes of high MMP11 expression group and low MMP11 expression group were primarily enriched in an extracellular matrix (ECM) organization (GO:0030198), extracellular structure organization (GO:0043062), collagen-containing ECM (GO:0062023), endoplasmic reticulum lumen (GO:0005788), ECM structural constituent (GO:0005201), ECM structural constituent (GO:0030020), protein digestion and absorption (hsa04974), ECM-receptor interaction (hsa04512) (**Figure 4G**). We selected the 50 genes with the most significant differences and performed PPIs analysis (**Figure 4H**). The results showed a direct link between EGFR and MMP11, and a possible pathway relationship between the two.

### 3.4 Correlation of MMP11 with immune cell infiltration in EGFR-mutant LUAD tumor tissue

**Figure 5:**
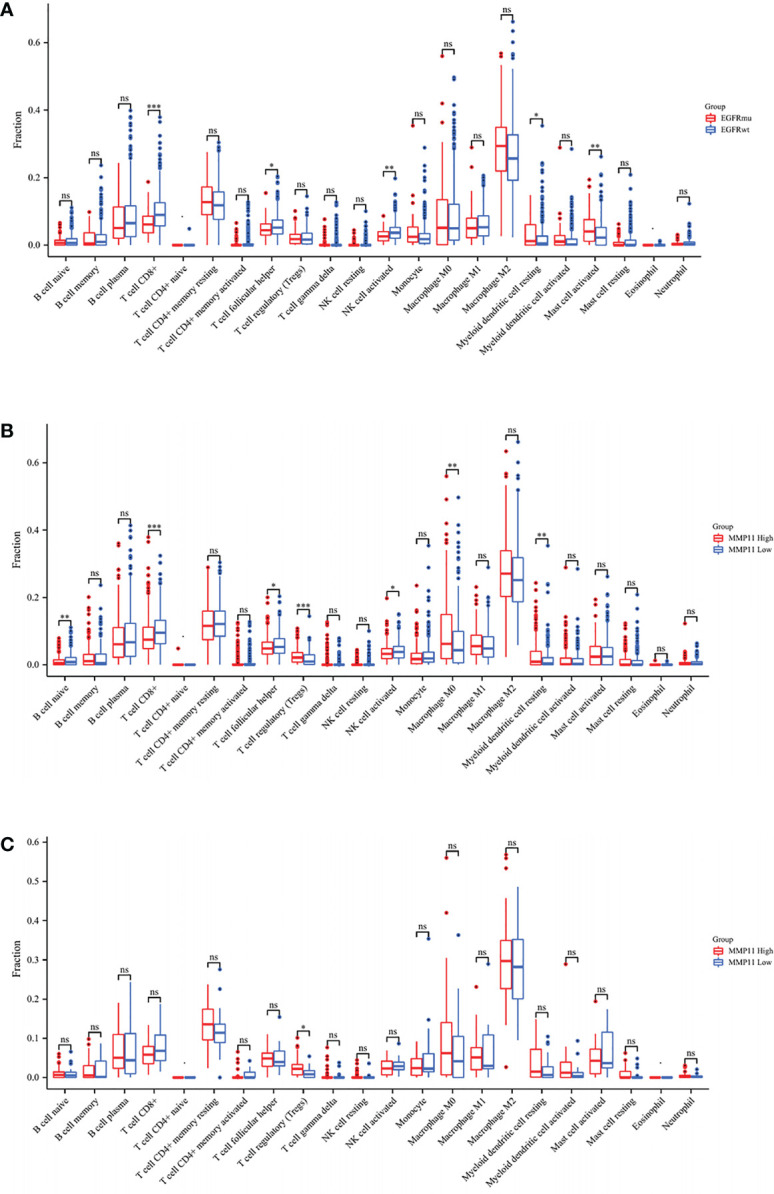
Differences in the infiltration of 22 immune cells in LUAD patients with TIME. **(A)** Results of high MMP11 expression group (red, n=258) compared to low expression group (blue, n=257) in TCGA-LUAD; **(B)** Results of EGFR mutation group (red, n=66) compared to a wild group (blue, n=445); **(C)** Results of high MMP11 expression group (red, n=46) compared to low expression group (blue, n=20) in EGFR-mutant LUAD; *P < 0.05; **P < 0.01; ***P < 0.001; ns, no statistical significance.

We next attempted to explore the relationship between MMP11 and the immune microenvironment from the perspective of immune infiltration. After missing values were deleted, LUAD samples were separated into two groups based on the median MMP11 mRNA expression border in the TCGA-LUAD database. The differences in immune cell infiltration levels between the high and low MMP11 expression groups were then investigated. Statistics are considered significant when P < 0.05. The high MMP11 expression group (red) consisted of 257 samples, while the low MMP11 expression group (blue) contained 256 samples. The results showed that in LUAD, B cell naive (P < 0.01), T cell CD8+ (P < 0.01), T cell follicular helper (P < 0.05), and NK cell activated (P < 0.05) were all reduced. T cell regulatory (Treg; P < 0.0001), macrophages M0 (P < 0.01), and myeloid dendritic cells resting (P < 0.05) were all increased in proportion (**Figure 5A**). We similarly analyzed the differences in immune cell infiltration of tumor tissue between the EGFR-mutant (red) and EGFR-wild (blue) groups in LUAD, showing that compared to the EGFR-wild group, patients in the EGFR-mutant group had a higher proportion of T cell CD8+ (P < 0.0001), T cell follicular helper (P < 0.05), NK cells activated (P < 0.05), Mast cells activated (P < 0.01) were reduced, and the percentage of macrophage M2 (P < 0.05), Myeloid dendritic cells resting (P < 0.05), and myeloid dendritic cells activated (P < 0.05) were all increased (**Figure 5B**). We next analyzed the differences in immune cell infiltration in the high and low MMP11 expression groups in EGFR-mutant samples (**Figure 5C**). We found that in EGFR mutant lung adenocarcinoma, Tregs cells were infiltrated to a greater extent in the MMP11 high expression group (red, n=46) compared to the MMP11 low expression group (blue, n=20) (P < 0.05). In contrast, there was no significant difference in the extent of infiltration of the remaining 21 immune cells (P < 0.05).

### 3.5 Multiplex immunohistochemistry reveals differences in the immune microenvironment of the high- and low-expression groups of MMP11

**Figure 6:**
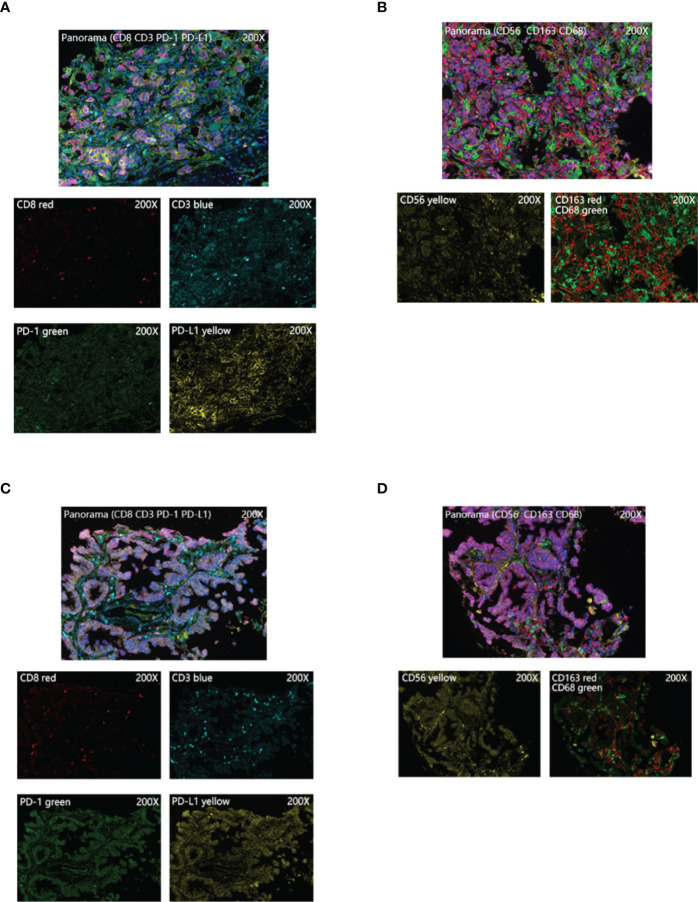
Typical images of multiplex immunohistochemistry. **(A, B)** Images of immune cell infiltration in the high MMP11 expression group of EGFR-mutant LUAD patients (200X); **(C, D)** Images of immune cell infiltration in the low MMP11 expression group of EGFR-mutant LUAD patients (200X).

**Figure 7:**
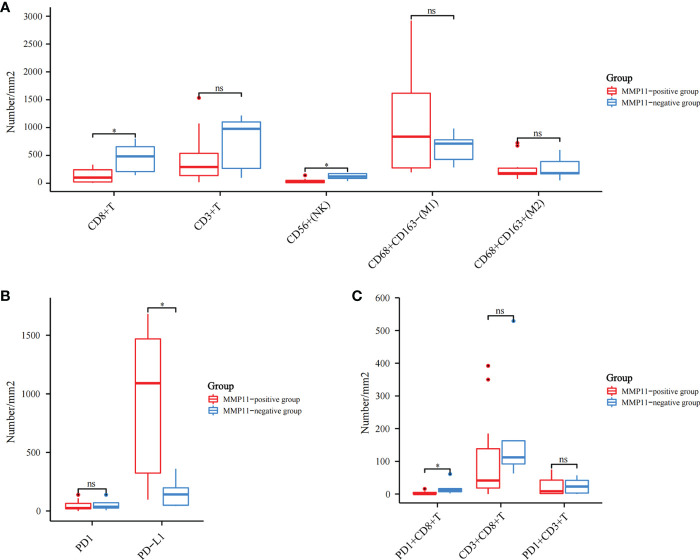
Different infiltration of immune cells in the tumor parenchyma of 17 EGFR-mutant LUAD cases. Red is the MMP11 positive expression group; blue is the MMP11 negative expression group; **(A)** Immune cell infiltration: CD3+ T cells, CD8+ T cells, NK cells, macrophages M1, macrophages M2; **(B)** The infiltration of different T-cell subtypes: PD1+CD8+ T cells, CD3+CD8+ T cells, PD1+CD3+ T cells; **(C)** The infiltration of CD3+CD8+ T cells and PD1+CD3+ T cells; *P < 0.05; ns, no statistical significance.

By further screening the obtained clinical EGFR-mutant LUAD samples, we acquired 17 samples subjected to multiplex immunohistochemistry and were divided into the MMP11 positive expression group and MMP11 negative expression group according to their MMP11 immunohistochemistry scores and were analyzed for clinical information (**Table 1**). There were 12 cases in the MMP11-positive expression group and 5 cases in the MMP11-negative expression group; however, there were non-significant variations between the two groups in gender, age, or TNM stages (P > 0.05). Using a microscope, immune cell infiltration images of 17 samples of tumor parenchymal immune cell infiltration, including immune cell (CD3+ T cells, CD8+ T cells, NK cells, macrophages M1, macrophages M2, PD1+CD8+ T cells, CD3+CD8+ T cells, PD1+CD3+ T cells), tumor cell PD-L1 expression, and immune cell PD-1 expression, and immune cell infiltration images of typical EGFR-mutant LUAD patients with high and low MMP11 expression groups were observed (**Figure 6**). We then divided the 17 samples into MMP11-positive (red, n=12) and MMP11-negative (blue, n=5) groups based on the results of the MMP11 immune score and compared the differences in immune infiltration between the two groups. The results suggested that the infiltration levels of CD8+ T cells and NK cells were lower in the MMP11-positive expression group than in the MMP11-negative expression group (P < 0.05), the infiltration levels of CD3 + T cells, macrophages M1, macrophage M2 and immune cell PD-1 showed nonsignificant differences (P > 0.05) (**Figure 7A**). The expression levels of tumor cell PD-L1 were higher in the MMP11-positive expression group than in the MMP11-negative expression group (P < 0.05), however, there was no significant difference in immune cell PD-1 expression between the two groups (P > 0.05) (**Figure 7B**). We further analyzed the immune infiltration of different T cell subtypes and found that the proportion of PD1+CD8+ T cells infiltrated was reduced in the MMP11-positive group compared to the MMP11-negative group (P < 0.05), while the extent of infiltration of CD3+CD8+ T cells and PD1+CD3+ T cells was not significantly different (P > 0.05) (**Figure 7C**).

**Table 1:** Baseline clinical data of 17 cases of EGFR-mutant lung adenocarcinoma.

Characteristics	MMP11-positive expression group (N=12)	MMP11-negative expression group (N=5)	Total (N=17)	P-value
Gender				
Female	5(41.67%)	2(40%)	7(41.18%)	1
Male	7(58.33%)	3(60%)	10(58.82%)	
age				
≥65	2(20.00%)	1(20%)	3(17.65%)	1
<65	10(80.00%)	4(80%)	14(82.35%)	
T stage				
T1	1(8.33%)	1(20%)	2(11.76%)	0.88
T2	2(16.67%)	1(20%)	3(17.65%)	
T3	2(16.67%)	1(20%)	3(17.65%)	
T4	7(58.33%)	2(40%)	9(52.94%)	
N stage				
N0	6(50%)	2(40%)	8(47.06%)	0.81
N1	4(33.33%)	3(60%)	7(41.18%)	
N2	1(8.33%)	0(0%)	1(5.88%)	
N3	1(8.33%)	0(0%)	1(5.88%)	
M stage				
M0	8(66.67%)	2(40%)	10(58.82%)	0.59
M1	4(33.33%)	3(60%)	7(41.18%)	

## 4 Discussion

Lung cancer genomics is better understood due to the rapid development of high-throughput sequencing technology, particularly in the area of gene targeting in LUAD. The treatment of patients with LUAD was significantly changed due to the sensitivity of EGFR mutations to EGFR-TKIs. LUAD is a tumor with frequently used “cancer driver” genes (28). Molecular-targeted therapies offer a promising alternative to traditional chemotherapy and radiotherapy as a kind of treatment (29, 30). Nevertheless, the effectiveness of EGFR-TKIs for LUAD with an EGFR gene mutation varies. In practice, EGFR-TKIs are ineffective for 20%–30% of NSCLC patients with mutant EGFR genes and offer no therapeutic benefit (31). Eventually, patients treated with first- or second-generation EGFR-TKIs acquire therapeutic resistance (32). Unfortunately, treatment with third-generation EGFR-TKIs drugs leads to resistance mutations (33, 34). Although third-generation EGFR-TKIs drugs, including axitinib, can effectively and selectively inhibit EGFR-TKI-sensitive and EGFR T790M-resistant mutations (32, 35). Treatment with third-generation EGFR-TKIs drugs also results in resistance mutations (36), and the resistance mechanisms are more complex and incompletely understood (37).

ICIs are designed to enhance the ability of immune system to kill cancer cells by blocking immune checkpoint signaling and restoring T-cell activity (38, 39). PD-1/PD-L1 antibodies have distinct treatment mechanisms and high efficacy to be the most widely used medications. Compared to conventional chemotherapy, ICIs can provide patients with lasting clinical benefits (40). However, not all patients can take advantage of them. In EGFR-mutant LUADs, PD-L1 expression is linked to early immune escape, primary treatment resistance, and fulminant progression (41–43). Clinical trials of EGFR-TKIs in combination with ICIs were terminated due to their severe toxicities (44). Atezolizumab in combination with carboplatin, paclitaxel, and bevacizumab significantly improved progression-free survival (PFS) in EGFR+/ALK+ NSCLC patients in the IMpower150 trial (45), compared to chemotherapy in combination with bevacizumab, indicating that EGFR-mutant LUAD patients are reversible regarding resistance to ICIs at various stages of the disease. In an *in vitro* experiment, a non-inflammatory tumor microenvironment was improved in patients with EGFR-mutant LUADs to increase their response to PD-1/PD-L1 (46). Therefore, research on the immune microenvironment may lead to the discovery of novel immunotherapy targets in EGFR-mutant LUAD patients.

According to studies, MMPs play a crucial role in the immunological microenvironment of tumors, regulating the body immune system and aiding in tumor immune escape (15). MMP11, also known as stromelysin-3, is a member of the MMPs family (17) and was first expressed in non-malignant fibroblast-like cells in the vicinity of breast cancer cells (16). MMP11 has unique features; it is processed in the cytoplasm and secreted as an active enzyme, while most MMPs are secreted as inactive zymogens. Furthermore, MMP11 does not hydrolyze the classical substrates of MMPs, such as laminin, fibronectin, and elastin. Finally, MMP11 has a relatively low potential to hydrolyze proteins compared to other MMPs. Due to its special structure, MMP11 has a unique role in tumor development compared to other MMPs molecules. Tumor cells can release MMP11 in an autocrine manner, actively altering the tumor microenvironment to interact with it to adapt to its malignant biological behavior (18). To describe the proteogenomic picture of LUAD in East Asia, Yi-Ju Che et al. (19) found that strong positive expression of MMP11 and MMP-7 was significantly associated with poor overall survival by analyzing tumor-related histological information from 103 Taiwanese LUAD patients. Those with high MMP11 gene expression levels had a lower prognosis than those with low expression levels, suggesting that MMP11 has a potential negative prognostic significance. However, a correlation between MMP11 and immune resistance after the mutation of the EGFR gene has not been unreported. Moreover, the core of immune resistance is immune escape. Therefore, we used MMP11 as a study factor to explore its relevance to the immune microenvironment in EGFR mutation-positive LUAD patients.

Differences in MMP11 expression in tumor tissues vs. normal tissues were explored using bioinformatic analysis techniques. The results revealed that MMP11 was highly expressed in various tumors, including LUAD. MMP11 can be expressed in different cancers through various enhanced expression pathways, while MMP11 expression is almost absent in normal tissues (17). To better verify this conclusion, we collected 59 LUAD tumor tissues for comparison with paracancerous tissues in the database of TCGA-LUAD. The results showed that MMP11 was significantly more expressed in LUAD tissues than in paraneoplastic ones. Next, we investigated the correlation between mutations in EGFR and MMP11 expression. Using differential analysis of gene expression in 511 LUAD samples with identifiable EGFR mutation status in the TCGA-LUAD database, we found that the mRNA level and MMP11 expression levels were increased in LUAD with EGFR gene mutations. This result suggests that MMP11 expression is associated with EGFR mutations. Since different EGFR mutation subtypes may have different biological behavioral functions, we further analyzed the differences in MMP11 expression levels in different EGFR mutation subtypes of lung adenocarcinoma (47). We found no significant difference in MMP11 expression between different EGFR mutation subtypes. In the analysis of MMP11 expression in each EGFR mutation subtypes group and EGFR-wild group, we found that the expression of MMP11 was not differential between the uncommon-type group and the EGFR-wild group, while the other subgroups showed high MMP11 expression levels compared to the EGFR-wild group. Classical EGFR mutant lung cancer with exon 19 deletions, exon 21 L858R and *de novo* exon 20 T790M mutations are associated with good response to tyrosine kinase inhibitors. In contrast, LUAD patients with uncommon EGFR mutations respond poorly to targeted therapy (48). One study suggested that patients carrying uncommon EGFR mutations had a similar immune microenvironment to EGFR-wild type patients and possibly benefitted from immunotherapy as much as the EGFR-wild patients (49). A retrospective study suggested that patients carrying uncommon EGFR mutations might have potential therapeutic sensitivity to PD-1 blockade (50). Accordingly, we consider that patients carrying non-classical mutations in EGFR at the levels of MMP11 expression are also similar to EGFR-wild patients, and whether this results in a similar immune microenvironment in these two groups of patients still requires following studies in LUAD.

In the exploration of the correlation between MMP11 and immune response, we found that MMP11 expression in lung adenocarcinoma did not correlate with cytotoxic T-cell level and the clinical benefit of ICB therapy regardless of whether EGFR is mutated or not. we first analyzed the correlation between MMP11 and TIDE core indicators in LUAD. TIDE uses a set of gene expression markers to assess 2 different mechanisms of tumor immune escape, including dysfunction of tumor-infiltrating CTL and rejection of CTL by immunosuppressive factors (26). Next, we analyzed the response to immunotherapy in the high and low MMP11 expression groups in EGFR-mutant LUAD using the TIDE algorithm score (27). The TIDE score uses a set of gene expression markers to assess two different mechanisms of tumor immune escape. The higher the TIDE score, the worse the immunosuppressive efficacy and the shorter the survival after receiving immunosuppressive therapy (27). The results showed high TIDE scores in the high MMP11 expression group in EGFR-mutant LUAD, high MMP11 expression may indicate a poor prognosis for immunotherapy. This suggests that high expression of MMP11 in EGFR-mutant lung adenocarcinoma suggests a poor immune response and has some unique significance.

To further analyze the possible pathways and mechanisms of MMP11 affecting immune response in EGFR mutant lung adenocarcinoma, we analyzed the differential genes between high and low MMP11 expression groups in EGFR mutant lung adenocarcinoma. Using differential gene analysis on samples from the high and low MMP11 expression groups in EGFR-mutant LUAD, we found that the gene upregulation trend was more obvious in the high MMP11 expression group in EGFR mutation-positive LUAD. The top 100 genes with the most significant alteration trend were enriched for the KEGG pathway and GO term. The results showed that in EGFR-mutant LUAD, the differential genes in the high MMP11 expression group and low MMP11 expression group were mainly enriched in the ECM organization, extracellular structure organization, collagen-containing ECM, endoplasmic reticulum lumen, ECM structural constituent, ECM structural constituent conferring tensile strength, protein digestion and absorption, and ECM-receptor interaction pathways. These pathways are mainly associated with the ECM, which is an important component of the tumor microenvironment and is also involved in immune regulation and affects the sensitivity of immunotherapy. The extra-tumoral matrix is an important component of the tumor immune microenvironment and is involved in tumor immunity (51, 52). We then analyzed the top 50 genes that were significantly different and the protein-protein network of EGFR with a view to understanding their association. The results suggest a possible pathway association between EGFR and MMP11. The pathway between EGFR and MMP11 in lung adenocarcinoma has not been investigated yet, but there are some results on the pathway related to MMP11 in other tumors. Yan Liu et al. found that GAST can regulate cell proliferation and metastasis related to gastric cancer prognosis through STAT3/MMP11 pathway (53). Bing Tan et al. demonstrated that MMP11 can activate the insulin-like growth factor 1 (IGF-1)/protein kinase B (AKT)/forkhead box protein O1 (FoxO1) signaling pathway in a series of preclinical mouse mammary tumor models, thereby affecting tumor metabolism and promoting cancer growth (54). Chao Su et al. found that MMP11 induced by IGF-1 may promote the proliferation and invasion of SGC-7901 cells through the JAK/STAT pathway by *in vitro* cellular assays and related molecular assays (55). In contrast, Ying Zhuang et al. identified the TGF-β signaling pathway as a potential downstream target of MMP11 by enrichment analysis of breast cancer sample data, further confirming that MMP11 knockdown can inhibit tumor proliferation and growth (56). There is also a link between MMP11 and EGFR, for example, increased EGFR phosphorylation results in upregulation of MMP11 expression that leads to increased migration, invasion and metastasis of breast cancer cells (57).Whereas in EGFR-mutant NSCLC cells overexpressing IGF-1R, inhibition of IGF-1R attenuates cell proliferation and VEGF production (58). Thus the mechanism regarding the role of EGFR and MMP11 in regulating immunity in lung adenocarcinoma is worthy of further exploration.

The tumor immunological microenvironment is crucial to immune response and immune escape in EGFR-mutant LUAD patients (59). The tumor immune microenvironment of EGFR mutant LUAD patients has reduced infiltration of CD8+ T lymphocytes and induced increased production of Treg, which is consistent with our results through database analysis. CD8+ T lymphocytes are effective factors in treating ICIs; a decrease in their percentage suggests that immunotherapy is ineffective and is associated with immune escape (60). Conversely, elevated Treg adversely affects the immune clearance of tumors and hinders effective immunotherapy (61). Deepali V Sawant et al. (62) found that Treg cell-derived IL-10 and IL-35 in the immune microenvironment against non-small cell lung cancer promoted BLIMP1-dependent CD8+ TILs depletion through a mouse model, and this synergistic effect limited effective antitumor immune potency. Accordingly, we analyzed the differences in the abundance of immune cell infiltration in the MMP11 high and low expression subgroups from the perspective of immune cell infiltration. We found that B cell naive, T cell CD8+, T cell follicular helper, and NK cell activated of patients in the high MMP11 expression group were decreased, and the proportions of T cell regulatory, macrophages M0, and myeloid dendritic cells resting were increased. As previously described, the decreased abundance of T cell CD8+ and the increased abundance of Treg contributed to immune escape, which is consistent with the analysis of EGFR-mutant group. Consequently, patients with LUAD in the high MMP11 expression group had immune cell infiltration patterns comparable to those with EGFR-mutant LUAD. Similarly, we discovered fewer NK cells activated cells in the EGFR-mutant group and the high MMP11 expression group. Without presensitization, NK cells have various strategies to eliminate cancer cells (63). Elizabeth L McMichael, using flow cytometry, found that activated NK cells can produce cytokines and chemokines with antitumor effects that recruit macrophages and T cells to inflammatory sites. This characteristic of NK cells shows less invasion and aids tumor immune escape. Therefore, we proposed that MMP11 could cause tumor cell immune escape in EGFR-mutant LUAD by modifying the immune microenvironment. However, in the EGFR mutant samples of the TCGA-LUAD dataset, only Tregs cells differed in the MMP11 high and low expression groups, and the infiltration level was higher in the high expression group than in the low expression group; the rest of the immune cell infiltration levels did not differ significantly. Considering that the tumor tissue in TCGA-LUAD includes both tumor parenchyma and tumor mesenchyme, the results may be influenced by the tumor simply.

Subsequently, in order to exclude the effect of tumor interstitial, we used multiplex immunohistochemistry to examine immune cell infiltration in the tumor parenchyma of EGFR-mutant LUAD, removing the influence of the tumor mesenchyme on the process. This technique is a technical breakthrough in three aspects: *in situ* labeling of tissue multiple targets, identification of multi-channel superimposed signals, and quantitative analysis of multi-metric morphology, in which the expression levels of multiple biomarkers (n ≥ 5) are detected *in situ* on a single tissue section, leading to identifying the phenotypic category, functional status, and their interrelationships of each cell on the tissue, and giving statistically significant cytometric data (64). This method is well suited to fully depict complex tissue microenvironment information, so it is called the tissue microenvironment panoramic analysis technique. After analysis, we found that samples with different MMP11 expression profiles in EGFR-mutant LUAD had different infiltration of immune cells in the tumor parenchyma. The infiltration levels of T cell CD8+ and NK cells were lower in the MMP11-positive expression group than in the MMP11-negative expression group. The expression levels of PD-L1 in tumor cells were higher in the MMP11-positive expression group than in the MMP11-negative expression group. Moreover, in EGFR-mutant LUAD, the proportion of PD1+CD8+ T cells infiltration was lower in the MMP11-positive group compared with the MMP11-negative group. PD-1 monoclonal antibodies work primarily by blocking and clearing PD-1-positive immunosuppressive state T cells, and T cell failure is thought to be one of the pathways of resistance to cellular therapies and depleted T cells are characterized by reduced cytokine production and expression of inhibitory receptors (such as PD-1, CTLA4 and LAG3) and the immunosuppressive enzyme CD39 (65, 66). Multiple studies have found that T cells expressing PD-1 depletion markers are enriched in CD8+ TILs that recognize the tumor reactivity of autologous tumor cells: the high abundance of CD8+ TILs expressing the failure marker PD-1 before or early in treatment predicts the clinical benefit of treatment with ICIs (67). The clinical samples we collected were all primary untyped treated sample tissues, so the low level of CD8+PD1+ T cells infiltration in the tumor parenchyma of the MMP11 positive expression group may suggest poor efficacy of ICIs. In the context of chronic antigen exposure, T-cell depletion is an inevitable corollary, and the criteria for assessing the efficacy of ICIs therapy differ between periods of T-cell depletion: Evidence of CD8+ T-cell depletion in pre-treatment and early treatment tumor samples predicts a clinically benign outcome of ICI treatment; depletion markers observed late in treatment and post-treatment predict an unfavorable clinical outcome (67, 68). This also suggests that dynamic observation of the infiltration level of depleted T cells in the immune microenvironment of EGFR-mutant LUAD patients with high expression of MMP11 may provide a basis for timing and monitoring the effect of immunotherapy in such patients. This is consistent with our previous analysis, suggesting that MMP11 may contribute to developing immune escape in EGFR-mutant LUAD patients by modulating immune cells. Accordingly, we propose that the immune microenvironment of LUAD differs with different levels of MMP11 expression and that high expression of MMP11 results in a downregulation of the abundance of immune cells that contributes to the antitumor effect and an upregulation of the abundance of immune cells that do not, both of which may make LUAD tumor cells more susceptible to immune escape.

ICIs primarily prevent tumor immunological evasion by inhibiting the relevant immune targets, which raises T-lymphocyte viability and has an anti-cancer impact. Immunotherapy is restricted in LUAD patients with EGFR mutations because they respond poorly to ICIs and are more likely to experience immune escape. In this study, using bioinformatic tools and simple tests, we investigated the relationship between MMP11 and the immunological microenvironment of this group of patients. The findings demonstrated that high levels of MMP11 in patients with EGFR-mutant LUAD are associated with a poor immune response and may control the tumor immune microenvironment, which fosters an immunosuppressive environment and aids in the immune escape of tumor cells. Consequently, MMP11 might be a cutting-edge immunotherapeutic target.

This study has several drawbacks. The limitations include a lack of in-depth analysis of the molecular pathway of MMP11 that results in immune escape in EGFR mutation-positive LUAD, a small number of clinical samples, and a lack of *in vivo* cell experiments to confirm the antitumor effects of MMP11 antibodies. Therefore, using molecular pathways and animal models, we will further explore the role of MMP11 in immune escape in patients with LUAD who have EGFR mutation. We want to provide fresh insights into the best course of treatment for these patients in the clinical context.

## Data availability statement

The raw data supporting the conclusions of this article will be made available by the authors, without undue reservation.

## Ethics statement

The studies involving human participants were reviewed and approved by the ethics committee of the Affiliated Hospital of Hebei University. The patients/participants provided their written informed consent to participate in this study.

## Author contributions

All authors were involved in experiments and manuscript editing. LB prepared the figures and wrote the manuscript. YS critically reviewed and made significant revisions to the manuscript. All authors contributed to the article and approved the submitted version.
