# Supplementary material for: MMP11 is associated with the immune response and immune microenvironment in EGFR-mutant lung adenocarcinoma
Source: Front Oncol. 2023 Jan 23;13:1055122. doi: 10.3389/fonc.2023.1055122 (PMC9900007; doi:10.3389/fonc.2023.1055122)
Supplement: Supplementary file 3 [file Table_1.docx]

Supplementary Material

**Supplementary Table 1 Inclusion and exclusion criteria of patients**

| Criteria for patient inclusion | Criteria for patient exclusion |
| --- | --- |
| Initial case of clinically confirmed adenocarcinoma of the lung | Patient has other malignant tumors at the same time |
| Genetic test results carrying EGFR mutant or EGFR wild-type cases | Tumor tissue for testing has a history of antitumor drug treatment prior to acquisition |
| A case of wax block of tumor tissue retained for pathological examination in the Department of Pathology of the Affiliated Hospital of Hebei University | External pathology testing or inability to provide sufficient amount of qualified tumor tissue specimens |
| Able to provide sufficient amount of qualified tumor tissue specimens | Other conditions deemed unsuitable for enrollment by the investigator |

**Supplementary Table 2 Correlation between MMP11 and clinical information of patients with LUAD (TCGA)**

| Characteristic | Low expression of MMP11 | High expression of MMP11 | p |
| --- | --- | --- | --- |
| n | 256 | 257 |  |
| T stage, n (%) |  |  | 0.566 |
| T1 | 90 (17.6%) | 78 (15.3%) |  |
| T2 | 131 (25.7%) | 145 (28.4%) |  |
| T3 | 24 (4.7%) | 23 (4.5%) |  |
| T4 | 8 (1.6%) | 11 (2.2%) |  |
| N stage, n (%) |  |  | 0.246 |
| N0 | 171 (34.1%) | 159 (31.7%) |  |

**Supplementary Table 2 Correlation between MMP11 and clinical information of patients with LUAD (TCGA) (continued)**

| Characteristic | Low expression of MMP11 | High expression of MMP11 | p |
| --- | --- | --- | --- |
| N1 | 41 (8.2%) | 54 (10.8%) |  |
| N2 | 36 (7.2%) | 38 (7.6%) |  |
| N3 | 0 (0%) | 2 (0.4%) |  |
| M stage, n (%) |  |  | 0.773 |
| M0 | 175 (47.4%) | 169 (45.8%) |  |
| M1 | 14 (3.8%) | 11 (3%) |  |
| Gender, n (%) |  |  | 0.270 |
| Female | 131 (25.5%) | 145 (28.3%) |  |
| Male | 125 (24.4%) | 112 (21.8%) |  |
| Race, n (%) |  |  | 0.405 |
| Asian | 5 (1.1%) | 2 (0.4%) |  |
| Black or African American | 28 (6.3%) | 24 (5.4%) |  |
| White | 190 (42.6%) | 197 (44.2%) | White |
| Pathologic stage, n (%) |  |  | 0.773 |
| Stage I | 138 (27.3%) | 136 (26.9%) |  |
| Stage II | 59 (11.7%) | 62 (12.3%) |  |
| Stage III | 39 (7.7%) | 45 (8.9%) |  |
| Stage IV | 15 (3%) | 11 (2.2%) |  |
| Primary therapy outcome, n (%) |  |  | **0.008** |
| PD | 41 (9.6%) | 27 (6.3%) |  |
| SD | 19 (4.5%) | 18 (4.2%) |  |
| PR | 6 (1.4%) | 0 (0%) |  |
| CR | 144 (33.8%) | 171 (40.1%) |  |
| Age, median (IQR) | 67 (60, 72) | 65 (58, 73) | 0.424 |
